# The Impact of the Interferon/TNF-Related Apoptosis-Inducing Ligand Signaling Axis on Disease Progression in Respiratory Viral Infection and Beyond

**DOI:** 10.3389/fimmu.2017.00313

**Published:** 2017-03-22

**Authors:** Christin Peteranderl, Susanne Herold

**Affiliations:** ^1^Department of Internal Medicine II, German Center for Lung Research (DZL), University of Giessen, Marburg Lung Center (UGMLC), Giessen, Germany

**Keywords:** interferon, interferon-stimulated genes, tumor necrosis factor-related apoptosis-inducing ligand, acute lung injury, innate immunity, influenza, respiratory syncytial virus, coronavirus

## Abstract

Interferons (IFNs) are well described to be rapidly induced upon pathogen-associated pattern recognition. After binding to their respective IFN receptors and activation of the cellular JAK/signal transducer and activator of transcription signaling cascade, they stimulate the transcription of a plethora of IFN-stimulated genes (ISGs) in infected as well as bystander cells such as the non-infected epithelium and cells of the immune system. ISGs may directly act on the invading pathogen or can either positively or negatively regulate the innate and adaptive immune response. However, IFNs and ISGs do not only play a key role in the limitation of pathogen spread but have also been recently found to provoke an unbalanced, overshooting inflammatory response causing tissue injury and hampering repair processes. A prominent regulator of disease outcome, especially in—but not limited to—respiratory viral infection, is the IFN-dependent mediator TRAIL (TNF-related apoptosis-inducing ligand) produced by several cell types including immune cells such as macrophages or T cells. First described as an apoptosis-inducing agent in transformed cells, it is now also well established to rapidly evoke cellular stress pathways in epithelial cells, finally leading to caspase-dependent or -independent cell death. Hereby, pathogen spread is limited; however in some cases, also the surrounding tissue is severely harmed, thus augmenting disease severity. Interestingly, the lack of a strictly controlled and well balanced IFN/TRAIL signaling response has not only been implicated in viral infection but might furthermore be an important determinant of disease progression in bacterial superinfections and in chronic respiratory illness. Conclusively, the IFN/TRAIL signaling axis is subjected to a complex modulation and might be exploited for the evaluation of new therapeutic concepts aiming at attenuation of tissue injury.

## Introduction

In 1957, Isaacs and Lindenmann (1) first recognized the potential of a soluble and probably cell-derived factor to combat influenza virus infection and named this factor interferon [(IFN) from latin *interferre*, to interfere]. Since then, three subgroups of IFNs have been defined, primarily by their differential receptor usage. While the groups of type I IFN and type III IFN comprise largely agents directly limiting pathogen spread by improving cellular counter measurements, IFN-γ, the sole type II IFN, has been mainly implicated in the modulation of innate and also adaptive immune responses (2, 3). Accordingly, type I and III IFNs are key signaling molecules in viral control, and lack of both signaling pathways results in increased viral loads and disease severity. Still, there is accumulating evidence that not only lack of an antiviral response but that also an unbalanced overshooting activation of IFNs contributes to an exaggerated inflammatory reaction, tissue injury, reduced proliferative capacity, and thus enhanced disease severity (Table 1). Especially in viral infections, this effect has not only been tracked down to IFN signaling in general but specifically to the exaggerated production of key effector IFN-stimulated genes (ISGs) (4). A prominent example is the TNF-related apoptosis-inducing ligand (TRAIL) that displays an ambivalent role in viral infection (5–7) (Table 1). Whereas first identified as factor produced by immune cells in non-respiratory infection (8, 9), TRAIL is now especially well studied in influenza A virus (IAV) infection, where it is released in high amounts from bone marrow-derived macrophages upon pathogen-associated molecular patterns (PAMPs) recognition and type I IFN production (10). Macrophage-released soluble TRAIL, but also membrane-bound cell-associated TRAIL, acts *via* distinct receptors on infected but also on non-infected, neighboring cells. In viral infection, its preliminary role is to drive infected cells into apoptosis to limit virus spread. However, studies performed within the last decade demonstrate that TRAIL’s antiviral activity seems to be outweighed by the functional and structural damage it induces not only in infected but also in bystander cells such as uninfected cells of the alveolar epithelium (10, 11). This process is not only relevant in promoting viral disease progression but has further implications in bacterial superinfection and probably also in chronic diseases. The recognition of the ambivalent role of IFN-driven signaling *in vivo* is a first important step to better understand disease progression and to envision novel treatment options for primary viral respiratory infection targeting distinct host-derived signaling mediators such as TRAIL.

**Table 1:** **Major effects of the interferon (IFN)/tumor necrosis factor-related apoptosis-inducing ligand (TRAIL) signaling axis on host cells in respiratory viral infection**.

	Effect	Virus	Reference
IFN	Virus control by antiviral interferon-stimulated genes induction	Influenza A virus (IAV)	(58–60)
		Coronaviruses (CoV)	(63, 64)
		Respiratory syncytial virus (RSV)	(62)
	e.g., *via*
	Interferon-induced transmembrane proteins	IAV, West Nile virus	(50, 51)
	Myxovirus resistance protein A	Vesicular stomatitis virus, IAV	(52–54)
	ISG20	IAV	(57)
	Restriction of immunopathology	IAV	(88)
		CoV	(63, 64)
		RSV	(62)
	Enhanced inflammatory response contributing to tissue damage, morbidity, and mortality	CoV	(76)
		IAV	(74, 95, 98)
		RSV	(67)
		Sendai virus	(73)
	Cell death induction, e.g., Bcl-2-associated X protein, caspase-8, Fas-associated protein with death domain, Fas ligand, and TNF-related apoptosis-inducing ligand (TRAIL)	dsRNA, polyI:C	(4, 110)
		IAV	(4, 5, 10, 115)
		Sendai virus	(110)

TRAIL	Virus control by apoptosis induction in infected cells	IAV	(6, 170, 171)
	Tissue injury by apoptosis of both infected and non-infected alveolar epithelial cells, lung macrophages	IAV	(5, 7, 10)
		RSV	(137)
	Necrosis of fibroblasts, dendritic cells, and epithelial cells	IAV	(146, 147, 168)
	Increased cellular infiltration	CoV	(175)
	Decreased expression of Na,K-ATPase, impaired epithelial fluid reabsorption	IAV	(11)

## From Pattern Recognition to ISGs—Basic Principles of IFN Signaling

### IFN Induction upon Virus Recognition

**Figure 1:**
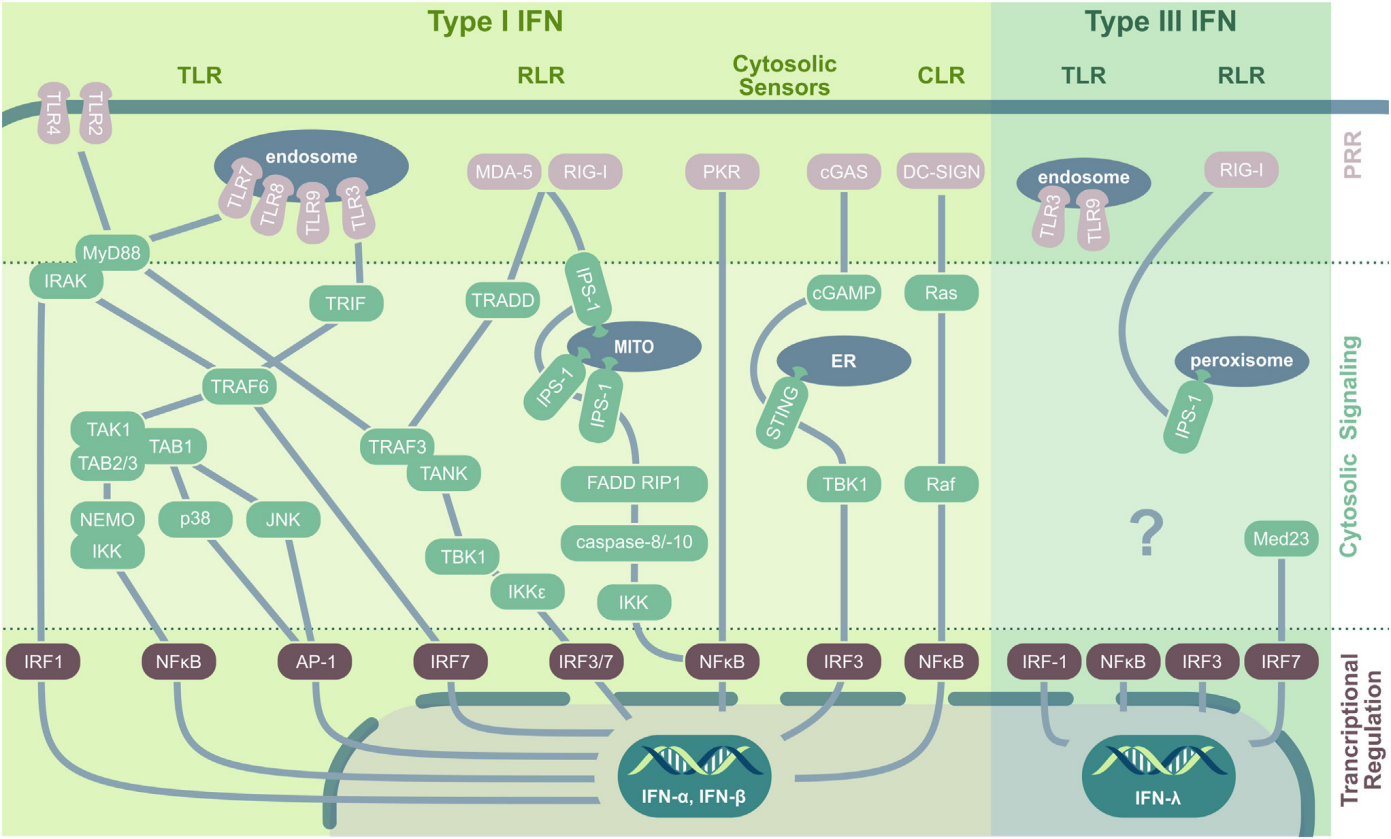
**PRRs and their downstream signaling pathways in virus-induced IFN induction**. In viral infection, type I IFNs are induced by TLR, RLR, CLR, and cytosolic nucleic acid sensors. Cell membrane-located *TLRs* ligate to viral envelope proteins (e.g., TLR2/herpes simplex virus), upon which they recruit MyD88. MyD88 interacts with IRAK kinase (IRAK-1, -2, or -4) that either directly activate IRF1 or interact with TRAF6, which induces IRF7 or assembles with TAK1. TAK1 forms a complex with TAB-1/-2 and -3 and subsequently either activates the MAPK kinases p38 and JNK, leading to AP-1 phosphorylation and nuclear translocation, or induces ubiquitination of NEMO followed by IκB degradation and NFκB activation. Endosomal TLRs recognizing viral nucleic acid and signal *via* the adaptor protein MyD88 (for TLR7/8/9) or interact with TRIF (for TLR3) followed by TRAF3, TANK, and TBK1 activation. TBK1 and IKKε then phosphorylate and activate IRF3 and IRF7. Additionally, TRIF can interact with TRAF6 to initiate TAK1 signaling. Both *RLRs*, RIG-I and MDA-5, recognize nucleic acid contents in the cytoplasm and stimulate the mitochondrial anchored IPS-1 for dimerization followed by TRADD recruitment that acts *via* TRAF3 on IRF3 and IRF7. Additionally, IPS-1 can interact with FADD and RIP1 to activate NFκB *via* IKK activated by caspase-8 and -10. Also *PKR* signaling results in NFκB activation and nuclear translocation. The dsDNA sensor *cGAS* produces cGAMP that activates ER-located STING that *via* TBK1 induces IRF3 translocation and type I IFN production. *CLRs* play a minor role in viral recognition; however, DC-SIGN activates the small GTPase Ras and Raf protein kinase, followed by NFκB activation. *Type III IFN* are induced by TLR3, TLR9, and *via* RIG-I and peroxisomal-resident IPS-1. Especially IRF1, but also NFκB, IRF3, and IRF7 are implicated in IFN-λ production, with the latter being stabilized by Med23. *Abbreviations*: TLR, toll-like receptor; RIG-I, retinoic acid-inducible gene; RLR, RIG-I-like receptors; CLR, C-type lectin receptors; PRR, pattern recognition receptor; MITO, mitochondrium; ER, endoplasmatic reticulum; IFN, interferon; MyD88, myeloid differentiation factor 88; IRAK, interleukin-1 receptor-associated kinase; IRF, IFN regulatory factor; TRIF, TIR-domain-containing adaptor protein-inducing IFN-β; TNF, tumor necrosis factor; TRAF, TNF receptor-associated factor; TAK, transforming growth factor β-activated kinase 1; TAB, TAK1-binding protein; NEMO, essential modulator; IKK, inhibitor-κB kinase; JNK, c-Jun N-terminal kinase; AP-1, activator protein 1; TANK, TRAF family member-associated NF-κB activator; TBK, TANK-binding kinase; MDA-5, melanoma differentiation antigen 5; TRADD, TNF receptor type 1-associated death protein; IPS-1, IFN-β promoter stimulation 1; FADD, Fas-associated protein with death domain; RIP1, receptor-interacting serine/threonine-protein kinase 1; PKR, protein kinase R; cGAS, cyclic GMP–AMP synthase; cGAMP, cyclic guanosine monophosphate–adenosine monophosphate; STING, stimulator of IFN genes; DC-SIGN, dendritic cell-specific ICAM3-grabbing non-integrin; AIM-2, absent in melanoma 2; Med23, mediator complex subunit 23; TRAF3, TNF receptor-associated factor 3.

It is a commonly accepted concept that—as Janeway (12) already proposed in 1989—immune activation toward invading pathogens is mounted upon recognition of PAMPs. PAMPs are evolutionary conserved biomolecules such as proteins, lipids, nitrogen bases, sugars, and complexed biomolecules such as lipoglycans that are essential to the survival of a given pathogen (13). PAMPs are recognized by distinct pattern recognition receptors (PRRs) that are germ-line encoded and—similar to PAMPs—usually show a high evolutionary conservation. The first recognized and probably most intensely studied family of PRRs are the toll-like receptors [TLRs; reviewed in Mogensen (14); Leifer and Medvedev (15)]. In viral infection, both host cell membrane-localized TLRs (TLR2, TLR4, detecting viral envelope proteins) and endosomal TLRs (TLR3, TLR7, TLR8, and TLR9, nucleic acid sensors) initiate signal transduction cascades leading to IFN production (Figure 1). TLR activation results in either myeloid differentiation factor 88 (MyD88) or TIR-domain-containing adaptor protein-inducing IFN-β (TRIF) recruitment that both trigger various downstream signaling events, eventually leading to IFN regulatory factor (IRF)3, IRF7, and NFκB nuclear translocation as well as MAP kinase and activator protein 1 (AP-1) activation (16, 17).

Similar to endosomal TLRs, the cytosolic retinoic acid-inducible gene (RIG)-I-like receptors (RLRs) are specialized to recognize viral nucleic acid contents and are central PRRs relevant to mount an antiviral response, providing resistance to most RNA (e.g., orthomyxoviruses) and some DNA (e.g., reoviruses) viruses [reviewed in Ref. (18, 19)]. Both melanoma differentiation-associated gene 5 (MDA-5) and RIG-I recognize dsRNA, 5′-triphosphate RNA, or the synthetic analog to dsRNA, polyI:C (20, 21). Both drive the dimerization of the mitochondria-associated adaptor protein IFN-β promoter stimulation 1 (IPS-1) (also named MAVS, VISA, CARDIF). A subsequently activated cascade including TRADD (TNF receptor type 1-associated death domain protein), TRAF3 (TNF receptor-associated factor 3), and TANK (TRAF family member-associated NF-κB activator) induces the phosphorylation of IRF3 and IRF7, resulting in type I IFN production (22). The third RLR, LGP2, so far has primarily been implicated to regulate RIG-I or MDA-5 as a cofactor; however, a recent study by Stone et al. (23) demonstrated a novel, non-redundant, and independent role of LGP2 in West Nile virus infection. Another class of PRR, the nucleotide oligomerization domain (NOD)-like receptors (NLRs), has mainly been implicated in bacterial recognition (24), still several NLRs are activated as well upon virus infection. Especially, NLRP3 is known to recognize RNA of different viruses including hepatitis C virus, measles virus, influenza, and vesicular stomatitis virus (VSV) (25–28), resulting in inflammasome formation and caspase-1-dependent activation of IL-1β and IL-18 (29–31). In addition, virus infections are sensed also by a structurally diverse group of viral RNA and DNA sensors residing in the cytoplasm. These include the cyclic GMP–AMP synthase that synthesizes the second messenger cGAMP. cGAMP in turn activates stimulator of IFN genes (STING), TANK-binding kinase 1, and IRF3, triggering IFN production (32–34). Moreover, STING itself acts as a PRR and has been implicated in DNA virus recognition including HSV, adenovirus, vaccinia virus, and papilloma virus and in sensing of retroviral RNA–DNA hybrids (35) and RNA viruses after being activated by RIG-I (36, 37). Another cytosolic nucleic acid sensor, PKR, is well known for its phosphorylation of eukaryotic initiation factor 2 α (eIF2α) in response to viral dsRNA. Phosphorylation of eIF2α results in its deactivation, host translational shut-off, and the limitation of viral replication. Of note, the PKR–eIF2α-driven inhibition of protein synthesis can contribute to an IPS-1-dependent IFN-β induction (38). Furthermore, PKR has been implicated in efficient type I IFN activation by TLR3 in response to dsRNA (39) and can mediate—at least partially—activities of IRF1 (40).

In addition to type I IFNs, also type III IFNs exert antiviral activity and are widely expressed after viral recognition, being produced by most cell types including epithelial, endothelial, fibroblast, and polymorphonuclear cells [reviewed in Ref. (41, 42)]. Like type I IFNs, type III IFNs are induced in viral infection by the PRR RIG-I as well as TLR3 and TLR9 and rely on the activation of the same transcriptional activators, including IRF3, IRF7, and NFκB. These observations initially led to the conclusion that type I and type III IFN comprised two completely redundant systems to induce ISGs in response to PAMP recognition. However, more recent data suggest distinct selection mechanisms for either type I or type III IFN expression. As such, IPS-1 specifically induces IFN-λ, but not type I IFN, when located at the peroxisomal membrane instead of the mitochondrial membrane in response to RIG-I activation by reovirus, Sendai virus, or dengue virus challenge (43). Interestingly, type III IFN induction is largely independent toward AP-1 translocation, which facilitates an instantaneous induction of IFN-λ after viral recognition, highlighting it as an important immediate factor driving innate microbial defense mechanisms.

### JAK/STAT-Dependent Induction of ISGs

Release of IFNs upon pathogen recognition is a highly conserved mechanism—found from teleost fish to insects and mammals—to prepare the surrounding cells as well as the host defense against the invading threat (44, 45). Whereas often high-level IFN production relies on specialized sentinel cells such as macrophages or dendritic cells (DCs), mostly all cells of the multicellular organisms are able to respond to at least one type of IFN by expression of respective receptors. Receptor binding then induces a signal transduction cascade relying on the Janus kinase (JAK) and Signal Transducer and Activator of Transcription (STAT), which results in efficient transcription of a plethora of different ISGs in infected as well as bystander cells (46, 47). IFNs engage a classical canonical signal transduction cascade employing JAK/STAT molecules after binding to their respective receptors. Herein, type I IFNs ligate to their common heterodimeric receptor consisting of the IFN-α receptor (IFNAR)1 and IFNAR2 subunits, whereas type III IFNs act *via* a interleukin-10 receptor 2 (IL-10R2)/IFN-λ receptor 1 (IFNLR1) heterodimer that to date has been reported to be restricted in its expression to epithelial cells (48). In type I IFN signaling, IFNAR engagement leads to the activation of the receptor-associated protein tyrosine kinases JAK1 and tyrosine kinase 2 followed by the recruitment or repositioning of already associated but elsewise latent cytoplasmatic transcription factors STAT1 and STAT2. Consequently, STAT1/STAT2 are phosphorylated on conserved tyrosine residues, they disassemble, undergo conformational changes enabling their heterodimerization as well as the exposure of a nuclear localization sequence. Subsequently, the STAT1/STAT2 heterodimer translocates into the nucleus where it interacts with the IRF9 to form the trimeric IFN-stimulated factor 3 (ISGF3). ISGF3 binds cognate DNA sequences, the IFN-stimulated response elements (ISRE), finally leading to ISG induction. Also type III IFN interaction with the IL-10R2/IFNLR1 receptor complex triggers STAT1/STAT2 heterodimerization, nuclear translocation, and ISGF3 assembly (49).

## IFNs in Acute Respiratory Viral Infection

Interferon signaling results in the induction of ISGs evoking different cellular responses against viral infection, both in infected as well as in non-infected cells, including direct antiviral, immune-modulatory, or cell death-inducing effects to enable an immediate and robust response to a pathogen challenge. Many ISGs directly interfere with viral replication on an intracellular level. Well-studied examples of antiviral ISGs comprise IFN-induced transmembrane proteins (IFITMs) effective in IAV, West Nile virus, and dengue virus infection (50, 51), the myxovirus resistance protein A (MxA) that interferes with VSV mRNA production and binds the IAV nucleocapsid to prevent nuclear translocation of viral genetic material (52–54), the 2-5-oligoadenylate synthase (OAS), which activates RNAse L triggering viral RNA degradation, or the PRR PKR, which besides activating the IFN response has a major impact on viral protein translation by inhibiting the eIF2α (55). More recently identified ISGs include the plasminogen activator inhibitor 1 that blocks IAV infection by inhibiting glycoprotein cleavage executed by extracellular airway proteases (56) or the antiviral ISG20 that limits IAV viral replication *via* its exonuclease activity most likely by interfering with the viral NP (57).

Accordingly, IFN pretreatment usually results in the establishment of an antiviral state that limits viral replication and spread from the start of infection and thus favors milder disease outcomes. IFN-α pretreatment has been demonstrated to limit viral spreading of seasonal IAV strains and thus decrease morbidity and mortality in mice, guinea pigs as well as ferrets (58–60). As shown by a study by Tumpey et al. (61), this effect can be attributed to the early induction of antiviral ISGs including MxA. Importantly, type I IFN pretreatment also dampens early replication of highly pathogenic avian influenza in ferrets (58). Also in respiratory syncytial virus (RSV) infection, treatment with recombinant IFN-α results in significantly decreased lung viral titers, alveolar inflammatory cell accumulation, and clinical disease in RSV-infected mice (62). In addition, respiratory infections caused by emerging coronaviruses (CoV) can be ameliorated by type I IFN pretreatment strategies. In an *in vivo* macaque model (*Macaca fascicularis*) of severe acute respiratory syndrome (SARS)-CoV infection, it could be demonstrated that pretreatment with pegylated IFN-α significantly diminished CoV replication and excretion and resulted in reduced pulmonary damage (63). Macaques also serve as a preclinical model for Middle East respiratory syndrome (MERS)-CoV, and similar to SARS-CoV, IFN-α in combination therapy with ribavirin reduces viral replication and severe histopathological changes (64).

In line, genetic alteration leading to an enhanced type I IFN signaling has been demonstrated to limit IAV-induced disease outcomes, as a recent study by Xing et al. (65) reported that deletion of TRIM29, a negative regulator of NEMO, which leads to NFκB induction and therefore enhanced type I IFN production, is protective *in vivo* in IAV-infected mice. Conversely, the genetic depletion of IFN signaling in IFN receptor-deficient mice can result in a lack of viral control, resulting in enhanced viral titers in different viral infections including RSV or IAV (66, 67). Still, it must be noted that this effect is often mild in IFNAR- or IFNLR-deficient animals, which is probably related to a certain redundancy between type I and type III IFN signaling in limiting viral spreading in epithelial cells (68). In contrast, IFNAR/IFNLR-double knockout or STAT1 knockout animals that are deficient in both type I and type III IFN signal transduction succumb more readily to infection due to excessive viral replication (69–71). *Vice versa*, mutations in key ISGs such as IFITM3 are associated with increased IAV disease severity in mice and humans (72).

However, IFN pretreatment and genetic loss-of-function approaches generally are not relevant to human respiratory virus-induced hospitalizations, where patients already present with ongoing respiratory infection and inflammation, and preclinical studies underline that type I IFN signaling in an already inflamed organ is rather detrimental and enhances tissue injury, and lack of type I IFN *in vivo* may even ameliorate disease outcome. Accordingly, in cases where the antiviral defense was not compromised (e.g., in animals with efficient type III IFN signaling) IFNAR-deficient mice infected with Sendai virus or IAV were reported to be more resistant to infection-induced morbidity and mortality (73, 74). Similarly, in Sendai virus *in vivo* infection, Wetzel et al. (75) showed that increased IFN-β levels in the lung homogenate correlates to increased morbidity and mortality, and also for SARS-CoV, a recent study demonstrates that high type I IFN induction in an already ongoing viral infection contributes to mortality in SARS-CoV-infected mice (76). Also for IAV infection, type I IFN application after infection has been proven to drive disease severity (74). Of note, the detrimental effects of type I IFNs were especially pronounced in mice lacking central antiviral factors, namely the IFIT protein in Sendai virus infection and MxA in IAV. Interestingly, Beilharz et al. (77) demonstrated that application of low doses of IFN-α reduces viral load, which to a certain degree led to attenuated disease progression, whereas high dose application of type I IFN contributed to morbidity (77). In line, high expression levels of ISGs have been shown to correlate to worse outcomes in ARDS patients (78). This observation corresponds to reports stating that the IFN threshold needed to induce antiviral ISGs—showing a beneficial effect in acute respiratory viral infection—is by at least 10-fold lower than the IFN dose necessary to trigger ISGs that show immunomodulatory, death-inducing, or anti-proliferative effects and thus can contribute to disease progression (79–82). Altogether, these data demonstrate that IFNs may significantly contribute to unbalanced inflammation and tissue injury during respiratory viral infection depending on expression levels and duration of IFN-related signaling events.

To date, the underlying mechanisms leading to the IFN-dependent enhanced disease progression are not fully understood but often result from a dysregulated IFN signaling response. One mode of action of IFN and IFN-stimulated ISGs is to stimulate negative feedback loops on IFN signaling. For example, suppression of JAK1 or STAT1 *via* specific phosphatases, expression of suppressor of cytokine signaling (SOCS)1 and SOCS3, or ubiquitination and endocytosis of the IFN receptors (83–86) desensitize cells to IFN signaling and allow recovery and the return to homeostasis after microbial challenge. As demonstrated by Bhattacharya et al. (87), the lack of IFNAR downregulation and thus the failure to initiate IFN-desensitization contributes to increased inflammatory signaling, extensive lung injury and, importantly, also impaired tissue regeneration (87). Moreover, IFNs are immunomodulatory and shape the specific responses of cells of the immune system, which has been implied to influence disease progression both positively and negatively. In a recent study, type I IFNs have been associated in the regulation of innate lymphoid immune cells (ILC)2 in IAV infection, where they—in concert with IFN-γ and IL-27—promote an ILC2-dependent restriction of immunopathology (88). Moreover, type I IFNs play an important role in stimulating the immune response driven by DCs; they stimulate the expression of MHC molecules as well as the co-stimulatory ligands CD80 and CD86 and thus activate T cell responses (89, 90). Additionally, ligand-driven activation of IFNAR enhances the proliferation of CD8 positive T cells, especially early in infection. However, late in infection, type I IFNs were also implied in decreasing T cell expansion upon SARS-CoV and arenavirus infection (76, 91), which might potentially be related to the above described desensitization upon prolonged IFN signaling and might be detrimental if initiated too early in infection. In line, Pinto et al. (92) reported an impairment of T cell responses upon type IFN induction in West Nile virus infection. In B cells, the lack of IFNAR has been demonstrated to result in enhanced release of neutralizing antibodies in IAV infection (93) implying a repressive role for type I IFN in B cell antibody production. However, immunization studies by Le Bon et al. (94) reported the necessity of IFNAR on B cells for efficient IgM and IgG production, underlining the need for further studies to understand the detailed effects of IFN-dosage and timing adaptive immunity activity upon respiratory viral infection.

Type I IFNs additionally induce the production of high levels of pro-inflammatory cytokines that have been closely linked to worsened outcomes of acute respiratory viral infection. Especially in IAV, disease severity and disease progression are linked with an overshooting, IFN-driven inflammatory response, in which further exogenous supplementation with type I IFN in fact correlates with increased morbidity and mortality (74, 95). In non-human primates, IAV infection with a highly pathogenic H5N1 isolate evokes a strong induction of type I IFN, resulting in severe lung injury by a necrotizing bronchiolitis and alveolotis (96). IFN levels in turn have been demonstrated to cause elevated pro-inflammatory cytokine levels after *in vivo* IAV infection and additionally, in human alveolar macrophages, the release of pro-inflammatory cytokines (e.g., MCP-1) are preceded by a robust type I IFN response (97). Importantly, also in human infection with H5N1, levels of pro-inflammatory cytokines are strongly elevated in bronchoalveolar lavage fluid, and cytokine levels have been associated with organ damage and worsened disease outcomes (98, 99). Still, it should be noted that due to strain differences in virus-elicited PRR activation and, importantly, IFN antagonism by the IAV non-structural (NS)1 protein, IFN levels and disease severity do not always directly correlate; actually, the extent to which NS1 can suppress the IFN response relates to prolonged viremia and thus can also be a determinant of virus pathogenicity both in human bronchial epithelial cells and in an *in vivo* model of IAV infection (100, 101). Alongside IAV, also in RSV infection the induction of high levels of pro-inflammatory cytokines has been directly related to type IFN, as RSV-infected but IFNAR-deficient mice presented with significantly diminished pro-inflammatory cytokine release, which translated into an attenuated disease course (67). Also in SARS-CoV, the late phase type I IFN induction relates to accumulation of inflammatory macrophage populations and elevated lung cytokine levels (76).

## TRAIL in Acute Respiratory Viral Infection—Limitation of Pathogen Spreading Versus Induction of Tissue Injury

### Cell Death Pathways in IFN Signaling

In addition to antiviral, immunomodulatory, and pro-inflammatory ISGs, IFN signaling results in the transcription and translation of cell death-inducing ISGs. In the context of viral infection, these factors provide a mode to block viral spreading and reinfection by killing those infected cells, in which the internal activation of antiviral ISGs is not sufficient to restrict viral replication. Thus, the infected cell is sacrificed to prevent the release of infectious progeny virions to limit viral spreading. However, especially in the lung, the disruption of the alveolar epithelial barrier by cell death of infected cells, but importantly also non-infected bystander cells induced by factors such as TRAIL, significantly contributes to worsened disease outcomes.

Controlled cell death or apoptosis can be induced by intrinsic and extrinsic signals. The intrinsic apoptosis pathway is initiated by diverse intracellular stimuli that influence the expression and activation of B cell lymphoma (Bcl)-2 family proteins that govern the permeabilization status of the outer mitochondrial membrane. Once cytochrome *c* is released from the mitochondria, it binds to the intracellular adaptor protein, apoptotic peptidase activating factor 1, forming the so-called apoptosome that in turn recruits pro-caspase-9 (102). Caspases (cysteine-aspartic proteases) exert their action by cleaving other proteins and substrates. Herein, initiator caspases such as caspase-8 and caspase-9 target other downstream caspases, whereas effector caspases, including caspase-3, -6, and -7, directly cause apoptosis by cleaving and thus inactivating or disassembling a vast array of cellular integral proteins and complexes (103). The extrinsic apoptosis pathway relies on an extracellular signal exerted by ligands of the tumor necrosis factor (TNF) receptor (TNFR) superfamily, including TRAIL, TNF-α, and Fas ligand (FasL) (104). Their ligation to their respective cell surface-expressed death receptors (DR) leads *via* the signal transmission by Fas-associated protein with death domain (FADD) to the activation of the initiator caspases-8 or -10, finally stimulating effector caspases including caspase-3 (105).

To date, several type I and type III IFN-induced, proapoptotic factors have been identified (106). Both caspase-4 and caspase-8 have been shown to be upregulated upon type I IFN signaling (4, 107); caspase-8 enhances the FADD-driven extrinsic apoptosis pathway, whereas the less-studied caspase-4 may promote pro-IL-1β cleavage and inflammasome-driven cell death (pyroptosis) in macrophages (108, 109). Chattopadhyay et al. (110) demonstrated that Sendai virus infection and polyI:C treatment resulted in Bcl-2-associated X protein (Bax) activation and apoptosis induction *via* one of the key transcription factors of IFN genes, IRF3. In addition IRF5 was reported to enhance TRAIL-dependent extrinsic apoptosis by nuclear translocation resulting in the translation of to date undefined factors that increase cell death upstream of caspase-8 activation (111). Furthermore, both RLRs, RIG-I and MDA-5, trigger the proteins Puma and Noxa that induce Bcl and thus activate the intrinsic mitochondrial apoptotic cascade (112). Also, PKR influences a cell’s susceptibility to apoptotic signals, as it was demonstrated to sensitize to the FADD/caspase-8 apoptosis pathway upon type I IFN signaling after challenge with IAV or dsRNA (4) and the OAS-RNAseL system has been suggested to contribute to IFN-α-related cell death induction, but the exact mechanisms remain to be elucidated (113). Finally, also two classical initiators of the extrinsic apoptosis cascade are induced as ISGs. Both FasL and its receptor Fas are upregulated on mRNA levels by IFN-α (114), and FasL was reported to be induced by type I IFN in IAV infection in the murine lung *in vivo* (115). Also, the proapoptotic factor TRAIL (or TNFSF10, Apo2L) is induced by IFN-mediated and ISGF3-executed transcriptional activation, as has been shown by Sato et al. (116), who revealed the presence of the ISRE sequence within the TRAIL promoter region (116). In IAV infection, TRAIL is released in high amounts from infected alveolar macrophages depending on a PKR- and IFN-β-driven autocrine signaling loop. Binding of IFN-β to macrophage-expressed IFNAR activates a JAK/STAT-dependent release of TRAIL, which then acts through its receptor DR5 on the alveolar epithelial cells (5, 10).

However, certain prerequisites may decrease the ability of a cell to undergo apoptosis, including a shortage in pro-caspase-8 availability, expression of cellular FADD-like IL-1β-converting enzyme-inhibitory proteins (c-FLIPs) that block FADD-driven caspase activation, inactivation, or degradation of FADD itself, or expression of CYLD, which acts as a receptor-interacting serine/threonine-protein (RIP)1 kinase de-ubiquitinase and thus stabilizes RIP1. However, in these cases IFN signaling can still promote a caspase-independent, programmed inflammatory cell death by activating the necroptosis pathway (117, 118). Necroptosis is induced by a complex formation by RIP1 and RIP3 kinases that activate both poly-ADP-ribose (PAR) polymerase 1 (PARP-1) and/or mixed lineage kinase domain-like (MLKL), leading to ATP depletion, calpain activation, PAR polymer accumulation or cell membrane permeabilization, and release of damage-associated molecular patterns, respectively [reviewed in Ref. (119, 120)]. Both a type I IFN-dependent JAK/STAT-driven activation of PKR as well as signaling by the PRR DAI (DNA-dependent activator of IRFs) initiates necroptosis *via* RIP1/RIP3 activation, respectively (117, 121).

Importantly, the activation of proapoptotic and pro-necroptotic pathways in respiratory infection can result in a structural disruption of the airway and the alveolar epithelial barrier, which is a major hallmark of respiratory disease and its progression to the acute respiratory distress syndrome (122, 123). In virus-induced lung injury, especially expression of TRAIL, which can initiate both apoptosis as well as necroptosis has been correlated with more severe outcomes.

### TRAIL-Induced Cellular Stress and Death Pathways

#### TRAIL and Its Receptors

As described earlier, TRAIL belongs to the superfamily of TNF ligands and has been reported to be inducible by both type I and type III IFNs. TRAIL has been found to be present in various cells of the immune system, among them natural killer (NK) cells, T cells, NK T cells, DC subsets such as IFN-γ-producing killer DCs and macrophages, and can be displayed in large amounts on the cell surface or be shed upon IFN- and/or pro-inflammatory cytokine signaling (124–126). In addition to cells of the immune system, fibroblasts have been shown to produce TRAIL after IFN-γ treatment or viral challenge. Also, club cells and the alveolar epithelium have been reported to produce TRAIL (127–130). Similar to other ligands of the TNF superfamily, TRAIL is a homotrimeric type II transmembrane protein with a conserved C-terminal extracellular domain that mediates receptor binding and can be cleaved by metalloproteinases to generate a soluble mediator (131). However, TRAIL can induce cell death also in its membrane-bound form, that is, similar to TRAIL expression levels and TRAIL shedding, upregulated by type I IFN (126). Direct cell-to-cell TRAIL–DR interactions have been demonstrated to play a role in macrophage, NK as well as CD4^+^ T cell-mediated induction of cellular death (132, 133).

In humans, five different binding partners for TRAIL are present: the membrane-bound DR4 (TRAIL-R1) and DR5 (TRAIL-R2) that both induce a proapoptotic signaling cascade, the membrane-bound anti-apoptotic decoy receptors (DcR)1 and DcR2, and the soluble interaction partner osteoprotegerin (134). In the murine system, only DR5 has been identified to ligate to TRAIL (135). In the human respiratory compartment, both DR4 and DR5 have been demonstrated to be present under steady-state conditions (136, 137). However, upon viral infection, cell-sensitivity to TRAIL-induced apoptosis is enhanced, which has been attributed to increased TRAIL receptor expression especially on infected cells, as DR levels are markedly increased in IAV-, adenovirus-, and paramyxovirus-infected cells in contrast to non-infected bystander cells (10, 138, 139). Of note, studies on the dependency of DR upregulation upon type I IFN signaling after IAV infection have yielded conflicting results in different strains of mice (10, 74), highlighting the complex interplay of IFN-induced cascades in a host- and tissue-specific context, whereas the exact virus- and host-specific mechanisms for DR regulation remain less well defined. Moreover, previous assumptions that also DcR expression would correlate with cell-sensitivity to TRAIL-induced cell death could not be experimentally verified (125).

#### TRAIL-Induced Signaling Cascades

**Figure 2:**
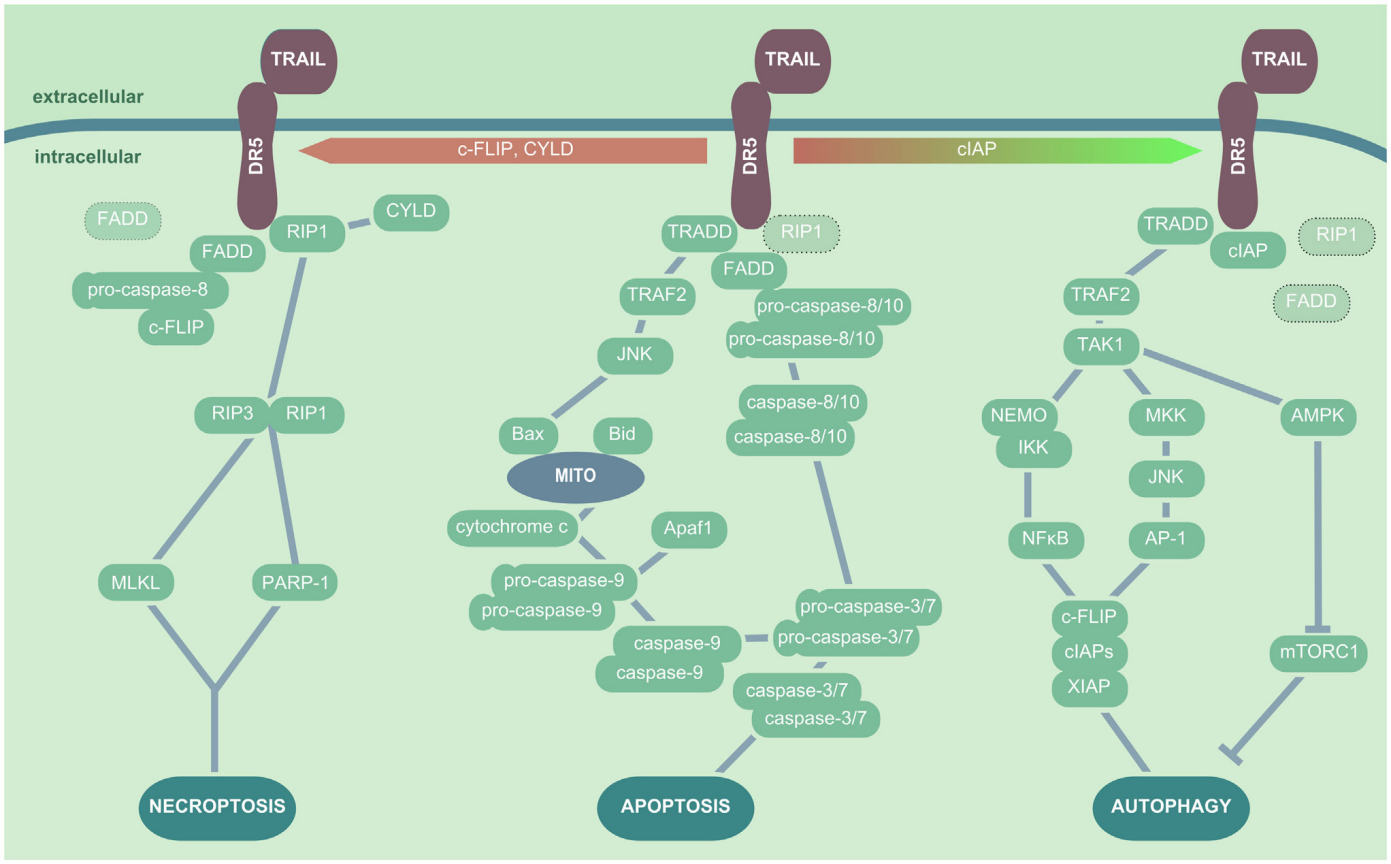
**TRAIL/DR5-mediated cellular signaling pathways**. In presence of RIP1, TRADD, and FADD, TRAIL ligation to DR5 results in apoptosis induction, which is initiated by recruitment of the pro-caspase-8 or -10 to FADD. These in turn activate the effector caspases-3 and -7, which leads to DNA fragmentation and apoptosis induction. In addition, TRADD can trigger a TRAF2- and JNK-dependent activation of Bax and subsequent release of mitochondrial cytochrome *c*, inducing the pro-caspase-9 activation. In the presence of CYLD, c-FLIP or absence of sufficient amounts of FADD or pro-caspase-8, TRAIL ligation to DR triggers the interaction of RIP1 and RIP3 kinase, which in turn cause cell death *via* induction of MLKL and/or PARP-1. In the presence of cIAPS, FADD is not recruited to DR5 upon TRAIL ligation, and TAK1 is activated by TRADD/TRAF2 interactions. TAK1 induces NEMO followed by IκB degradation and NFκB activation, as well as MKK and JNK activation leading to AP-1 nuclear translocation; both events promote the production of cytoprotective factors such as XIAP, cIAPs, and c-FLIP. Additionally, TAK1 triggers AMPK activation and thus mTORC inhibition, which results in enhanced autophagic activity. *Abbreviations*: AP-1, activator protein 1; TNF, tumor necrosis factor; TRAIL, TNF-related apoptosis-inducing ligand; DR5, death receptor 5; RIP1, receptor-interacting serine/threonine-protein kinase 1; TRADD, TNF receptor type 1-associated death protein; FADD, Fas-associated protein with death domain; TRAF, TNF receptor-associated protein; JNK, Janus kinase; Bax, Bcl-2-associated X protein; c-FLIP, cellular FADD-like IL-1β-converting enzyme-inhibitory proteins; RIP, receptor-interacting serine/threonine-protein; MLKL, mixed lineage kinase domain-like; PARP-1, poly-ADP-ribose (PAR) polymerase 1; cIAP, cytoprotective factors including inhibition of the autophagic machinery; XIAP, X-linked inhibitor of apoptosis protein; AMPK, AMP-activated protein kinase; mTORC, mammalian target of rapamycin complex; TRAF2, TNF receptor-associated protein 2; MKK, mitogen-activated protein kinase.

Tumor necrosis factor-related apoptosis-inducing ligand ligation to the proapoptotic receptors DR4 or DR5 triggers a trimerization of the receptors. Subsequently, depending on additional stimuli, presence or absence of adaptor molecules or inhibitory proteins, different signaling pathways can be activated (Figure 2). In the classical TRAIL-dependent extrinsic apoptosis induction, the proteins RIP, TRADD, and FADD are subsequently recruited to the DR cytoplasmic domain upon TRAIL ligation (140, 141). These factors and the proapoptotic DRs all share a cytoplasmic death domain (DD), which is lacking or truncated and thus inactive in the DcR. The DD plays a central role in the concerted formation of the death-inducing signaling complex (DISC). DISC formation exposes a second functional domain of FADD, the death effector domain that is directly able to recruit pro-caspase-8 and pro-caspase-10. How exactly DISC formation induces caspase activation is still under debate. The most probable scenarios include either an autocatalytic cleavage of caspase-pro-domains enabled by the spatial proximity between pro-caspases (generated by their recruitment to DISC), by pro-caspase dimerization, or by pro-caspase conformational stabilization (125). Removal of the pro-domain of caspase-8 and caspase-10 results in the activation of the effector caspases-3 and -7, which cleave DNA fragmentation factor 45 and lead to apoptosis (142, 143). Moreover, TRAIL-binding to DR4 and DR5 can induce the JNK either *via* caspase-8 or recruitment of TNF receptor-associated protein 2 (TRAF2) to the DISC complex, which results in the activation of the intrinsic apoptotic cascade by Bax-dependent mitochondrial cytochrome *c* release (144). In addition, TRAIL signaling is also able to induce necroptosis by both activating the RIP1/RIP3 kinase downstream effectors PARP-1 and MLKL, contributing to epithelial cell death and tissue injury (145–147).

It has become apparent in recent years that TRAIL signaling is closely linked to induction of autophagy, a process generally associated with the blockade of apoptosis and necrosis. Indeed, autophagy has been reported to improve cellular survival in cell stress by catabolic removal of cytoplasmic long-lived proteins and damaged organelles. It also contributes to viral clearance and the transfer of viral material to endosomal-/lysosomal-located TLR7 or MHC class II compartments for the activation of adaptive immunity (148). Several studies outline that TRAIL ligation to DR5 can result in a TRAF2-dependent activation of TAK1 (MAP3K7) that has been attributed a central role in TRAIL-induced autophagy activation (149). TAK1 modulates the IKK-dependent translocation of NFκB, and it also induces JNK activation *via* mitogen-activated protein kinase. Both events lead to expression of autophagy-related factors including inhibition of the autophagic machinery (cIAP)1, cIAP2, X-linked inhibitor of apoptosis protein, and c-FLIP (150, 151). Especially, c-FLIP has been associated with desensitization of cells to TRAIL-induced apoptosis, favoring autophagy-related cascades (152). Another study revealed that upon TRAIL signaling the AMP-activated protein kinase (AMPK) is activated. AMPK in turn inhibits the mammalian target of rapamycin complex 1 that itself is an inhibitor of autophagy, thus the activation of the autophagic machinery is promoted (153). The decision if TRAIL signaling results rather in necroptotic or apoptotic cell death or in activation of autophagy seems to be dependent on the presence of cIAPs that promote RIP kinase ubiquitination and degradation (146), but also on the balance between active caspases and autophagic proteins such as Beclin-1 (154, 155). This suggests a scenario where autophagy is activated as cell protective mechanism until cell stress—as executed by enhanced TRAIL signaling or additional viral infection—increases over a threshold to favor cell death induction. Accordingly, as TRAIL signaling is not restricted to infected cells, excessive cell death activation might be limited by autophagy induction in non-infected bystander cells. However, autophagy is not only related to cell survival but can also positively affect apoptosis and induce—even if the exact mechanisms are still under debate—autosis, the autophagy-related cell death, another mode of TRAIL to trigger cell death (156, 157). Of note, autophagy activation needs to be placed into its virus-specific context, as some viruses, including Dengue virus, poliovirus, and Coxsackie B virus (158), can exploit autophagic pathways for their own replication and thus promote apoptosis and tissue injury.

### TRAIL in Acute Respiratory Viral Infection

As discussed above, TRAIL is a potent activator of cell death. However, its signaling outcomes can differ largely depending on its delivered form (e.g., membrane-bound versus soluble), the availability of DRs on the target cell membrane, alternate intracellular pathways that might be activated and finally the pathogen itself, as it might exploit TRAIL-induced pathways for its own survival and replication. In acute respiratory infection, TRAIL signaling is often part of an IFN-driven overshooting inflammatory reaction that promotes unspecific tissue injury and thus disease severity by increasing functional and structural changes in infected but also non-infected cells, as will be outlined below.

#### Influenza A Virus

The release and effects of TRAIL have been especially well studied in IAV infection in the last decade. Earlier studies reported that within 3 days after infection, bronchial, bronchiolar, and alveolar epithelial cells undergo apoptosis (159). This early induction of cell death is mainly attributed to direct apoptosis induction by the virus itself, as IAV actively promotes apoptosis for efficient viral replication (160). Herein, the viral NS1 and PB-F2 proteins not only play a crucial role (161, 162) but also the viral M2 protein has been implicated in this process as it inhibits autophagy in infected cells (163). In addition, our own data revealed that later in IAV *in vivo* infection, the recruitment of bone marrow-derived macrophages *via* the CC chemokine receptor type 2 (CCR2)–CC-chemokine ligand 2 (CCL2) axis significantly contributes to alveolar cell apoptosis and structural damage of the alveolar epithelium (5). Studies by Wurzer et al. (164) had previously demonstrated that IAV promotes the production of proapoptotic factors in an auto- and paracrine fashion *via* NFκB transcriptional activation by IAV (164). Subsequently, Brincks et al. (6) elucidated that human peripheral blood mononuclear cell treated with IAV released TRAIL and that increased TRAIL levels correlated with type I as well as II IFN induction. Additionally, TRAIL sensitivity was increased in influenza virus-infected cells. In line, our investigations could elucidate that IAV triggers a PKR-dependent translocation of NFκB that results the production of type I IFNs. These in turn induce, *via* ligation to the IFNAR receptor complex, expression and shedding of TRAIL by bone marrow-derived macrophages (10). In addition, Davidson et al. (74) demonstrated that type I IFN application to IAV-infected mice increased morbidity and lung injury, which could be attributed to both DR5 and TRAIL upregulation inducing epithelial cell apoptosis. Importantly, Högner et al. also reported that the IAV strain used in these studies, A/PR8 (H1N1), which is highly pathogenic for mice, induced an approximately 800-fold induction in macrophage TRAIL expression, whereas the lower pathogenic virus A/X-31 (H3N2) only stimulated TRAIL by a factor of eight. Of note, the relation between TRAIL induction and IAV strain-specific pathogenicity also translates to the highly pathogenic avian H5N1 IAV, causing severe pneumonia in mice as well as in humans (165, 166). Moreover, human infection with both the highly pathogenic H5N1 as well as the pandemic 1918 H1N1 IAV strains are characterized by a massive influx of mononuclear phagocytes into the alveoli, which is correlated with extensive alveolar epithelial cell apoptosis (97, 167). Additionally, macrophages gained from bronchoalveolar lavages of patients presenting with ARDS caused by the pandemic H1N1/2009 virus strain showed high surface expression and release of TRAIL (10). Another recent report demonstrates that in highly pathogenic avian influenza, in addition to macrophages also the alveolar epithelium might be involved in causing elevated levels of TRAIL in the alveolar space (130). Besides its role in apoptosis, TRAIL signaling upon IAV infection has also been implicated in the induction of necroptosis in fibroblasts, DCs, and lung epithelial cells (146, 147, 168). Rodrigue-Gervais et al. (146) demonstrated that lack of cIPA2 promotes RIP3 kinase-mediated necroptosis in response to TRAIL—but also the proapoptotic factor FasL—released from hematopoietic cells. This contributed to severe lung epithelial degeneration and increased mortality, even though viral control was not compromised. Nogusa et al. (147) further elucidated that IAV-induced necroptosis depends on RIP3 kinase activation of MLKL, and that RIP3 kinase deficiency, similar to cIAP2-deficiency, increased IAV-susceptibility *in vivo*.

In IAV infection, as mentioned earlier, DR5 expression is elevated on infected alveolar epithelial cells, but not in non-infected cells *in vivo*, which might impact on TRAIL susceptibility to apoptosis induction (10). However, both infected as well as neighboring bystander cells were found to be targeted for apoptosis induction by macrophage-released TRAIL. Nonetheless, we could recently show that specifically in non-infected cells within the IAV-infected lung, TRAIL severely compromises the function of the ion channel Na,K-ATPase, which was mediated *via* induction of the stress kinase AMPK (11), thereby potentially revealing a cross-link to TRAIL-induced autophagic cell stress pathways in bystander cells both *in vitro* and *in vivo*. The TRAIL-induced and AMPK-mediated downregulation of the Na,K-ATPase, a major driver of vertical ion and fluid transport from the alveolar airspace toward the interstitium, resulted in a reduced capacity of IAV-infected mice to clear excessive fluid from the alveoli. Thus, TRAIL signaling contributes to intensive edema formation, a hallmark of disease in virus-induced ARDS (123). Notably, this effect of TRAIL on Na,K-ATPase expression was induced independently of cell death pathways elicited by caspases, as treatment of cells and mice with a specific caspase-3 inhibitor diminished apoptosis in alveolar epithelial cells but still allowed for the reduction of the Na,K-ATPase (11). Conclusively, treatment of IAV-infected mice with neutralizing antibodies directed against TRAIL or the abrogation of recruitment of TRAIL^+^ bone marrow-derived macrophages inhibited apoptosis of both non-infected and bystander cells. Thus, lung leakage due to loss of alveolar barrier function was reduced, whereas alveolar fluid clearance capacity was enhanced, resulting in reduced edema, improved survival, and outcome upon IAV challenge *in vivo*. However, TRAIL has also been shown to be upregulated on NK, DC, and on CD4^+^ and CD8^+^ T cells after IAV infection (169). Studies by Brincks et al. demonstrated that especially CD8^+^ T involved in cytotoxic T cell responses toward IAV and drive IAV-infected cells into apoptosis *via* TRAIL, thus contributing to efficient virus clearance (6, 170). In addition, both FasL and TRAIL are involved in DC-mediated CTL activation and cytotoxicity against IAV-infected cells (6, 171). Furthermore, studies showed delayed viral clearance upon neutralizing anti-TRAIL antibody administration (169, 172). Our data, however, demonstrate that the transfer of TRAIL-deficient bone marrow into irradiated wild-type mice, resulting in loss of TRAIL production by bone marrow-derived macrophages upon IAV infection, does not impact on the capacity to fully clear viral particles from the lung at day 7 after infection, suggesting that other compensatory mechanisms are recruited to guarantee viral clearance (10). Taken together, in IAV infection, TRAIL acts both as an important mediator of infected cell killing but particularly as a detrimental factor contributing to tissue injury and impaired inflammation resolution when released in excessive amounts by recruited immune cells.

#### Respiratory Syncytial Virus

Respiratory syncytial virus is an important cause of respiratory tract infections especially in children worldwide. Generally, there seem to be virus-elicited anti-apoptotic mechanisms active in the lung epithelium, as RSV-infected primary human airway cells show a minimal cytopathic effect (173). However, several cell lines including small airway cells, primary tracheal-bronchial cells, and A549 and HEp-2 showed increased expression of TRAIL and its ligands DR4 and DR5 in an *in vitro* RSV infection model (174). Moreover, soluble TRAIL released from leukocytes was elevated in the bronchoalveolar lavage fluid of patients with RSV-associated respiratory failure, suggesting that similar to IAV, TRAIL contributes to RSV-induced epithelial injury and disease progression (137).

#### Coronaviruses

Also in CoV respiratory tract infection, TRAIL levels, but less so FasL, have been reported to be markedly elevated (175, 176). For SARS-CoV that presents with a severe damage to both the upper and lower respiratory tract (177), especially DCs respond with a strong induction of TRAIL production, which was suggested to correlate to increased cellular lung infiltrations present in SARS-CoV patients (175). Interestingly, SARS-CoV infection drives cells into apoptosis by a PKR-driven but eIF2α-independent pathway (178), which might—similarly as seen in IAV infection—suggest a PKR-induced and autocrine/paracrine executed activation of apoptosis.

Also MERS-CoV, which causes pneumonia and respiratory failure, has been demonstrated to induce profound cell death within 24 h of infection, irrespective of viral titers produced by the infected cells. However, type I IFN expression is strongly reduced in MERS-CoV in comparison to seasonal human CoV in *in vitro* infection models, including human monocyte-derived macrophages, Calu-3, and human lung fibroblasts (179, 180), which might also dampen downstream TRAIL induction. Therefore, the exact mechanism by which MERS-CoV promotes cell death remains to be investigated.

## The IFN/TRAIL Axis in Bacterial Superinfection after Viral Injury

Recurrently, viral infections of the respiratory tract are followed by outgrowth of colonizing Gram-positive bacteria that aggravates the course of illness. This is well documented for IAV, where “super” infections with *Streptococcus pneumoniae* and *Staphylococcus aureus* are the most frequent and increase viral pneumonia-associated morbidity and mortality (181). During the 1918 IAV pandemic, bacterial pneumonia was evident in most cases (182) and also during the recent 2009 H1N1 pandemic, coinfections were a relevant factor for severe disease in a young patient population without comorbidities (183). Interestingly, virus-induced elevation of the type I IFN response levels might promote secondary bacterial outgrowth by several mechanisms [reviewed in Ref. (184)]. In line, it has been repeatedly demonstrated that lack of type I IFN signaling results in better bacterial clearance and increased survival rates in IAV- and *S. pneumoniae-*superinfected mice (185–187). Herein, IFN-induced apoptosis induction as well as depletion or impaired recruitment of lymphocyte subsets necessary for bacterial control play a critical role (188, 189). Bacterial clearance from the lung has been reported to rely on sufficient phagocyte generation, recruitment, and survival. Type I IFN has been demonstrated to cause apoptosis in bone marrow-derived granulocytes, affecting the numbers of recruited neutrophils (189), but also to impair expression of the cytokines CXCL1 (or KC) and CXCL2 (or MIP-2), thus inhibiting neutrophil recruitment to the lungs with severe effects on survival of superinfected mice (185). A recent report by Schliehe et al. (190) elucidated the mechanistic background for impaired CXCL1 expression and secretion and demonstrated that type I IFNs activate the histone methyltransferase Setdb2, which in turn represses the Cxcl1 promoter and thus impairs neutrophil recruitment and bacterial clearance. Moreover, type I IFN production decreases CCL2 production, thus inhibiting macrophage recruitment, which as well has been reported to have detrimental effects on bacterial clearance and disease progression in bacterial superinfection after viral insult *in vivo* (186). In addition, type I IFNs also impair γδ T cell function and IL-17 release, which was shown to increase susceptibility to *S. pneumoniae* superinfection after IAV challenge (187). Also in *S. aureus* pneumonia, a robust type I IFN response is correlated to excessive morbidity and tissue injury (191). In a model of polyI:C, *S. aureus* (methicillin-resistant strain, MRSA) superinfection, polyI:C treatment prior to bacterial infection enhanced type I IFN levels and decreased bacterial clearance and survival (192). Furthermore, Shepardson et al. (193) demonstrated that late type I IFN induction rendered mice more susceptible to secondary bacterial pneumonia in a model of IAV–MRSA superinfection.

Only limited data are available on a direct role of TRAIL in respiratory disease progression due to bacterial superinfections. In a model of IAV–*Haemophilus influenza* infection, neither deficiency for CC chemokine receptor type 2, inhibiting bone marrow-derived macrophage recruitment, nor deficiency of Fas or TNFR1 impacted outcome (194). Yet, during *S. pneumoniae* single infection, early cell death of macrophages is thought to limit an exuberant inflammatory reaction and accordingly, a study by Steinwede et al. (195) revealed that neutrophil-derived TRAIL limits tissue injury by inducing cell death in DR5-epressing lung macrophages in bacterial mono-infection (195). In contrast, in the IAV–*S. pneumoniae* superinfection mouse model, IAV-induced TRAIL has a detrimental effect on overall mortality (7), as TRAIL-induced epithelial injury enhanced bacterial outgrowth of *S. pneumoniae*—administered at day 5 after IAV infection—markedly. Importantly, administration of anti-TRAIL neutralizing antibodies enhanced bacterial control by the host organism. Thus, the activation of IFN/TRAIL-mediated signaling in viral infection has detrimental implication for outcome of secondary bacterial infection following viral insult, rendering the IFN/TRAIL signaling axis an interesting therapeutic target not only in respiratory viral infections but also in complicating bacterial superinfection.

## IFN/TRAIL Axis in Chronic Lung Diseases

An increasing number of reports connect progression of chronic respiratory disease to acute respiratory virus infection or proapoptotic signaling events. In fact, TRAIL has been reported to be a critical determinant for promoting the development of chronic lung disease in early life (196); targeting TRAIL by genetic deletion or neutralizing antibody application in early-life respiratory infections ameliorated infection-induced histopathology, inflammation, as well as emphysema-like alveolar enlargement and lung function. Furthermore, TRAIL was also shown to play a role in the development of allergy and asthma. TRAIL is not only elevated in the sputum of asthmatic patients but has also been reported to be highly expressed in an experimental mouse model of asthma, where it induces CCL20 secretion by bronchial epithelial cells, thus promoting T_H_2 cell responses and airway hyperreactivity (197).

In COPD, acute exacerbations driven by viral and bacterial infection are a major factor increasing both mortality and morbidity, and both influenza and *S. pneumoniae* have been identified among the most common causes of COPD exacerbations (198). Indeed, primary bronchial epithelial cells isolated from subjects with COPD show an impaired production of type I IFN (199), which has been implied in the enhanced susceptibility of COPD patients to respiratory infections; however, even in absence of high IFN induction, both an abnormally elevated loss of alveolar epithelial cells due to apoptosis as well as elevated TRAIL and DR5 levels were reported (200), implying a possible link between viral/bacterial induction of TRAIL and acute exacerbations in COPD. TRAIL induction has also been directly linked to cigarette-smoke exposure, a common cause of COPD, and TRAIL deficiency resulted in decreased pulmonary inflammation and emphysema-like alveolar enlargement *in vivo* (201). Moreover, increased levels of both TRAIL and DR5 were associated to impaired lung function and increased systemic inflammation in human COPD patients (202). While alveolar epithelial cell death is closely connected to idiopathic pulmonary fibrosis (IPF), TRAIL and its receptors DR4 and DR5 in AEC were shown to be upregulated in IPF lungs (129). Also, in pulmonary arterial hypertension virus infection is considered to be a possible risk factor (203), and pulmonary hypertension has been reported to be a side effect of prolonged treatment with type I IFN (204, 205). In line, TRAIL has been closely linked to disease progression in pulmonary hypertension. TRAIL has been found to be increased within pulmonary vascular lesions of patients with pulmonary hypertension (206) and also in a mouse model of hypoxia-induced pulmonary hypertension, levels of soluble TRAIL correlated with right ventricular systolic pressure, right ventricular hypertrophy, and pathologic alterations (33, 34). Importantly, neutralizing antibody-treatment against TRAIL showed positive effects on survival while reducing pulmonary vascular remodeling (207). Notably, the extent to which infection-induced TRAIL release causes or exacerbates chronic lung disease or in how far TRAIL production in chronic lung diseases affects susceptibility to respiratory viral and complicating bacterial infection remains to be elucidated.

## Outlook: Therapeutic Concepts Targeting TRAIL in Acute Respiratory Viral Infection

Respiratory viral infections are major causative agents for lung injury and ARDS; however, in many cases antivirals are not sufficient to limit disease (208). Besides the fact that most viruses are subject to strong selective pressures that favor quickly evolving, drug-resistant virus variants, recent advances in understanding the processes that contribute to tissue injury and ARDS highlight a crucial role of immune-related, IFN-driven events. Therefore, novel therapeutic strategies often aim to improve the outcome of severe respiratory infection by modulating host cell responses; however, to date, clinical trials trying to improve severe viral infections or ARDS outcomes by targeting host pathways have not resulted in approval of new drugs (122).

Of note, for establishment of such therapies it has to be considered that the timing and intensity of induction and amplification as well as of dampening and termination of the IFN-driven immune response needs to precisely match the pathogen- and organ-specific requirements of a given infection. A non-controlled regulation of these processes may lead to either an unrestricted pathogen spreading or, on the other extreme, to an overshooting inflammatory response, including the increased production of pro-inflammatory and proapoptotic mediators, elevated levels of recruited immune cells, and/or aberrant repair processes. Notably, both too low and too high levels of IFN-induced effects facilitate disease progression with a possible increase of fatal outcomes in ARDS patients (78). Accordingly, preclinical *in vivo* studies of IFN-directed therapies yielded seemingly adverse results, depending on the context, timing, and dosage of IFN modulation. However, in multiple settings of acute respiratory viral infection, studies demonstrate that an exaggerated signaling derived from type I IFN in an already inflamed tissue contributes to worsened outcomes, and importantly, might favor secondary bacterial superinfection [e.g., Ref. (75, 76, 209)]. Interestingly, Davidson et al. (209) demonstrated that type III IFN release upon influenza challenge—in contrast to type I IFN induction—does not trigger an unbalanced inflammatory response that critically contributes to respiratory disease progression *in vivo*, highlighting it as a possible therapeutic option in IAV-induced lung injury. Most likely, this effect derives from the lack of the IFN-λR1/IL-10R2 receptor complex, but presence of IFNAR, on immune cells, including bone marrow-derived macrophages. Nonetheless, other reports identify IFN-λ as a driver of macrophage polarization to an inflammatory M1 phenotype (41) that has been attributed to further promote an overshooting inflammatory response, highlighting the need for further studies of type III IFN biology in pathogen-associated disease progression.

As generally IFN-directed therapeutic approaches target various downstream signaling events that might both act beneficially as well as detrimentally on viral replication and pathogenesis, a further approach is to address specific ISGs that primarily show detrimental effects on disease progression. As outlined above, TRAIL or its downstream signaling events might comprise a suitable target for adjunct therapies in addition to antivirals. Accordingly, our own data in a preclinical mouse model of IAV infection demonstrate a clear benefit of the systemic application of neutralizing antibodies against TRAIL at days 3 and 5 postinfection for lung injury, morbidity, and mortality (10, 11). Targeting TRAIL as a major determinant of disease severity in respiratory viral infections including IAV, but also RSV and CoV, may yield therapeutic approaches that are superior to IFN-directed strategies, as they seemingly do not bear the risk of compromising host defense. Yet, it should be thoroughly excluded that blocking TRAIL-induced cell death of infected cells will not lead to an overwhelming viral spreading, especially as reports on viral loads upon TRAIL inhibition in preclinical models of IAV are controversial (6, 10, 170). Accordingly, additional studies are needed to understand how and to which extent virus-infected cells can be killed or viral spreading can be controlled by other means in the absence of TRAIL. Moreover, targeting pathways and signaling hubs downstream of TRAIL/DRs, such as AMPK (11), in a well-timed and lung compartment-specific way, may open new therapeutic avenues but requires more detailed preclinical studies on efficacies and side effects. A valid approach might be the use of a combination therapy of such a treatment together with a classical antiviral drug therapy limiting viral replication; however, exact dosage, timing, kinetics, and application routes remain to be defined.

## Author Contributions

CP and SH performed bibliographic research and drafted the manuscript.

## Conflict of Interest Statement

The authors declare that the research was conducted in the absence of any commercial or financial relationships that could be construed as a potential conflict of interest.
